# Neutrophil Extracellular Traps in Systemic Lupus Erythematosus Stimulate IgG2 Production From B Lymphocytes

**DOI:** 10.3389/fmed.2021.635436

**Published:** 2021-04-12

**Authors:** Roberta Bertelli, Francesca Schena, Francesca Antonini, Daniele Reverberi, Sara Signa, Nicoletta Pedemonte, Alessandro Consolaro, Marco Gattorno, Simone Negrini, Francesca Pupo, Stefano Volpi, Gian Marco Ghiggeri

**Affiliations:** ^1^Laboratory of Molecular Nephrology, Division of Nephrology and Transplantation, Scientific Institute for Research, Hospitalization and Health Care (IRCCS) Giannina Gaslini Institute, Genoa, Italy; ^2^Laboratory of Human Genetics, Scientific Institute for Research, Hospitalization and Health Care (IRCCS) Giannina Gaslini Institute, Genoa, Italy; ^3^Centre for Autoinflammatory Diseases and Immunodeficiencies, Scientific Institute for Research, Hospitalization and Health Care (IRCCS) Giannina Gaslini Institute, Genoa, Italy; ^4^Core Facilities Flow Cytometry and Cell Imaging Lab, Scientific Institute for Research, Hospitalization and Health Care (IRCCS) Giannina Gaslini Institute, Genoa, Italy; ^5^Molecular Pathology Unit, Ospedale Policlinico San Martino, Genoa, Italy; ^6^Department of Neurosciences, Rehabilitation, Ophtalmology, Genetics and Maternal and Children's Sciences (DINOGMI), University of Genoa, Genoa, Italy; ^7^Complex Operative Unit (UOC) of Medical Genetics, Scientific Institute for Research, Hospitalization and Health Care (IRCCS) Giannina Gaslini Institute, Genoa, Italy; ^8^Pediatric Rheumatology Clinic, Scientific Institute for Research, Hospitalization and Health Care (IRCCS) Giannina Gaslini Institute, Genoa, Italy; ^9^Department of Internal Medicine, Clinical Immunology and Translational Medicine Unit, Policlinico San Martino, University of Genoa, Genoa, Italy; ^10^Division of Nephrology, Dialysis, Transplantation, Scientific Institute for Research, Hospitalization and Health Care (IRCCS) Giannina Gaslini Institute, Genoa, Italy

**Keywords:** lupus nephritis, autoimmunity, NETosis, T-bet, IgG2, naïve B cells

## Abstract

Circulating autoantibodies of IgG2 isotype predominate in Systemic Lupus Erythematosus (SLE) and concur to the development of the renal lesions characteristic of Lupus Nephritis (LN). Anti-dsDNA and anti-histones IgG2, together with anti-podocyte proteins (i.e., α-enolase) are the major autoantibodies in serum and renal glomeruli of LN patients. The mechanisms underlying autoantibody formation and isotype switching in SLE and LN are unknown. A major issue is how DNA/histones are externalized from cell nucleus, driving the autoimmune response. Neutrophil Extracellular Traps (NETs) have been recently identified as crucial players in this context, representing the main source of DNA and nucleosome proteins. A second key point is what regulates IgG2 isotype switching: in mouse models, T-bet transcription factor has been described as essential for IgG2a class switch. We hypothesized that, in SLE, NET formation is the key mechanism responsible for externalization of autoantigens (i.e., dsDNA, histones 2,3, and α-enolase) and that T-bet is upregulated by NETs, driving, in this way, immunoglobulin class switch recombination (CSR), with production of IgG2 autoantibodies. The data here presented show that NETs, purified from SLE patients, stimulate *ex vivo* IgG2 isotype class switch possibly through the induction of T-bet. Of note, we observed a prominent effect of NETs on the release of soluble IgG2 in SLE patients', but not in healthy donors' B cells. Our results add important knowledge on the mechanisms of IgG2 class switch in SLE and contribute to further elucidate the role of NETs in LN pathogenesis.

## Introduction

Systemic Lupus Erythematosus (SLE) is an autoimmune disease characterized by heterogeneous clinical manifestations, varying from minimal symptoms, such as fever and small joint pain, to severe organ lesions (1). Lupus Nephritis (LN) is the most frequent and severe complication of SLE, occurring in almost 50% of cases and frequently leading to renal failure (2). It is characterized by antibody deposition in glomeruli, with typical patterns that vary from modest localization in mesangium to diffuse sub-epithelium deposition, with subsequent activation of a complement-mediated inflammatory cascade (3). The clinical association of different types of autoantibodies with LN has been extensively studied in the past (4); investigators have, in particular, focused on the correlation of renal lesions with specific autoantibodies, mainly anti-dsDNA, anti-nucleosome, and anti-histones (anti-H1, anti-H2A, and anti-H3) (5–10). More recently, the analysis of potentially pathogenic antibodies, micro-eluted from glomeruli, has been completed; beside confirming the glomerular localization of anti-dsDNA, anti-histones (H2A, H3, H4) and anti-C1q antibodies (11), the presence of antibodies against two cytoplasmic proteins, i.e., anti-α enolase and anti-AnnexinA1 (12), characterized by the prevalence of IgG2 isotype (13, 14), was also shown. For their predominance, IgG2 autoantibodies have been defined as “nephritogenic”.

Two main issues involved in the generation of autoantibodies vs. intracellular/intranuclear antigens remain unclear: one concerns the way how antigens are externalized so as to trigger the autoimmune cascade, the second question concerns what regulates, in SLE patients, the production of IgG2, that usually is not the dominant subclass in the immune response. For several reasons, Neutrophil Extracellular Traps (NETs) have attracted the interest of researchers, since they may explain a part of these unresolved issues. NETosis is a sort of cellular death, in which DNA and histones are externalized from neutrophils, and form a sort of net where pathogens are entrapped and killed (15): a significant feature of NETs is the massive presence, beside DNA and histones, of α-enolase, the major autoantigen in SLE (16). Moreover, NETs are potent inducers of autoimmunity (17, 18) and stimulate both memory B cells to produce IgG autoantibodies and plasmacytoid dendritic cells (pDCs) to produce type 1 Interferon (19, 20). An autoreactive B cell population, overexpressing CD11c (an integrin involved in antigen presentation by B cells), is highly expanded in SLE patients and has been associated with renal disease severity (21, 22). Human CD11c^high^ cells are able to differentiate *in vitro* into antibody secreting cells (ASC) and produce IgG upon stimulation with inflammatory cytokines (23). In murine models of Lupus, the transcription factor T-bet has been shown to be overexpressed in this B cell subset and it is essential for the production of pathogenic IgG2a (24, 25), associated with the development of lupus-like disease (26).

On the basis of the above findings and given the well-established role of IgG2 autoantibodies in mice and their exclusive presence in SLE and LN patients, we hypothesized that, also in humans, NETs might act directly on the differentiation of autoreactive B cells into IgG2 secreting cells. Considering the crucial role of T-bet in the early phases of autoimmunity, we also attempted to determine whether NETs could directly induce T-bet expression in human naïve B cells. In particular, we focused on the evaluation of the immunogenic activity of NETs isolated from LN patients, because of their unique proteomic composition.

## Materials and Methods

### Blood Samples and Patients

Blood samples were obtained from 14 healthy donors (HDs) and 13 SLE patients. Patients 1–10 developed SLE at pediatric age and were on long term follow-up within Rheumatology Unit of Giannina Gaslini Institute, Genoa; patients 11–13 received SLE diagnosis at adult age and were on follow-up at S. Martino Hospital, Genoa. In both cases disease diagnosis was made according to the American College Rheumatology Systemic Lupus classification criteria as revised by the Systemic Lupus International Collaborating Clinics (SLICC).

Informed written consent was obtained from each individual before participation, upon approval of the local Ethic Committee. Clinical features are shown in Supplementary Table 1.

### LN-NETs Preparation

Polymorphonuclear cells (PMNs) were isolated from fresh heparinized blood samples of patients with LN, using the Ficoll/Dextran separation method (14) and plated onto 24-well plastic dishes at the concentration of 3 × 10^6^ cells/well. NETosis was induced by 30 nM Phorbol Myristate Acetate (PMA) stimulation, as previously described (27). Cells were then incubated with 15 U/ml Micrococcal DNase (Cayman, Ann Arbor, MI, USA) for 30 min at 37°C and the reaction was stopped by the addition of 5 mM EDTA. Samples were cleared from cellular debris by 13,000 rpm centrifugation. The molecular size of DNA fragments, obtained by DNase digestion, was assessed by 2% agarose gel electrophoresis. DNA and protein contents of NET extracts were determined by means of Nanodrop measurements and Bradford assay, respectively. Samples were then stored in aliquots at −80°C.

### Purification of B Cells and NET Uptake Assessment

Peripheral blood mononuclear cells (PBMCs) were obtained from HDs buffy coats or from heparinized blood samples of Lupus patients, through Lymphoprep (Sentinel Diagnostic, Milan, Italy) density gradient centrifugation. B cells were purified from PBMCs by positive selection with CD19+ Microbead Isolation Kit (Miltenyi Biotec Bergish Gladbach, Germany). CD19+ cells were plated onto 48-well plastic dishes, at the concentration of 5 × 10^6^ cells/well in RPMI 1640 medium (BioWhittaker, Lonza, Belgium), containing 10% Bovine Serum (FCS, Defined, Hyclone, Euroclone, Milan, Italy), 2 mM l-Glutamine, 10 mg/ml non-essential amino acids, 1 mM pyruvate, 50 U/ml penicillin, 50 U/ml streptomycin (Gibco, Grand Island, NY, USA), 5 × 10^−2^ M 2-mercaptoethanol (Sigma-Aldrich, St. Louis, USA) and supplemented with 4,000 U/ml Interleukin 2 (Proleukin, Chiron Corp., Emeryville, CA, USA), 2.5 μg/ml CpG2006 (5′-tcgtcgttttgtcgttttgtcgtt-3′; TIB Molbiol, Genoa, Italy) and 1.5 μg/ml F(ab')_2_ anti human IgM, IgA, IgG (Jackson Immunoresearch Europe, West Grove, PA, USA). Cells were cultured overnight; NET extracts (3 μg/ml protein concentration) were added after 16 h and were incubated for 2 h at 37°C. Following 2 washes in PBS, cells were stained with FITC-conjugated anti-human CD20 monoclonal antibody (Miltenyi Biotec) and plated onto polylysine coated high-quality 96-well clear bottom black plates, suitable for confocal microscopy. After fixation and permeabilization with BD Cytofix Cytoperm Buffers (BD Biosciences, San Jose, CA, USA), according to the manufacturer's instructions, cells were further stained with an Alexa 647-conjugated anti-human Myeloperoxidase (MPO) monoclonal antibody (Dako, Agilents Technologies, Santa Clara, CA, USA) that specifically evidenced the cellular binding and localization of NET fragments (25). Cells were then washed, and cell nuclei were counterstained with Hoechst 33342. Imaging was performed using Opera Phenix (PerkinElmer, Waltham, MA, USA) high-content screening system. Wells were imaged in confocal mode, using a 40X water-immersion objective. Alexa 647 signal was laser-excited at 640 nm and the emission wavelengths were collected between 650 and 760 nm. Excitation and emission wavelengths for visualization of Hoechst 33342 signal were 405 and between 435 and 480 nm, respectively.

### *In vitro* Proliferation and Differentiation of Naïve B Cells in the Presence of NET

Naïve B cells were enriched by cell sorting (BD-FACSAria instrument, BD Biosciences, San Jose, CA, USA), following the staining with Pe-Cy7-conjugated anti-CD19, FITC-conjugated anti-CD24 and APC-conjugated anti-CD38 monoclonal antibodies (Biolegend, San Diego, CA, USA). To identify the population of interest, we used the gating strategy described in Supplementary Figure 1. The sorted naïve B cell population had a purity of around 99.5%. IgD and CD27 expression on our gated CD19+ CD24- CD38^low^ evidenced the presence of a minimal contamination of memory B cells (CD27+ IgD-) that were ranging from 6.6 to 11.4% (data not shown).

Enriched naïve B cells, from SLE patients and HDs, were labeled with 0.5 μM 5-(and-6-)-carboxyfluorescein diacetate, succinimidyl ester (CFSE) (Molecular Probes, Eugene, OR,) for 8 min at room temperature, plated onto 96 multi-well plates and activated with the following stimuli: 2.5 μg/ml CpG2006, 5 μg/ml, F(ab')_2_ anti human IgM, IgA, IgG (Jackson Immunoresearch Europe, West Grove, PA, USA) and 4,000 U/ml IL-2 (Proleukin, Chiron Corp., Emeryville, CA). LN-NET extracts were added to cultures on day 5, at the concentration of 0,5 μg/ml protein.

At day 7 cells were harvested, washed in PBS and labeled with CD19 PE-Cy7, CD27 APC-Cy7, and CD38 APC for 30 min at +4°C; proliferation rate (analyzed by CFSE diluition assay) and plasmablasts (CD27+, CD38^high^) percentages were determined in viable (propidium iodide negative) CD19^+^ cells, using a BD-FACSCanto Flow Cytometer and Kaluza Software (Beckman Coulter Life Sciences, IN, USA).

### *In vitro* Culture of Naïve B Cells and Determination of Soluble IgG Subclasses

Soluble IgG levels were determined, by ELISA assay (28), in culture supernatants collected at day 7 from enriched naïve B cells, in the same culture conditions described above. Briefly, Dynatech M129A ELISA plates (Corning Costar, Glendale, AZ, USA) were coated with 10 μg/ml goat anti-human IgG or anti-human IgG2 antibodies (Southern Biotech, Birmingham, AL, USA), diluted in 0.1 M Phosphate Buffer (pH 9.6), and left overnight at 4°C. Plates were then washed with PBS + 0.05% Tween and saturated for 2 h with PBS + 10% FCS at room temperature (RT). Serial dilutions of cell supernatants and of IgG isotype standards were incubated for 2 h at RT. Plates were washed four times with PBS + 0.05% Tween and incubated with 0.5 μg/ml sheep HRP-conjugated anti-human IgG1 or anti-IgG2 secondary antibodies for 2 h at RT. After 4 washes with PBS + 0.05% Tween, the reaction was developed with 75 μl of 3,3′,5,5′-tetramethylbenzidine (TMB) substrate (Sigma-Aldrich, St. Louis, MO, USA) and it was stopped after ~5 min by 2 N Sulfuric Acid. Absorbance values were read at 450 nm using a microplate reader (Eppendorf, Milan, Italy).

### Analysis of Intracellular T-Bet Expression

*Ex vivo* T-bet expression was evaluated on CD19+ cells, obtained from previously isolated frozen PBMCs of 4 healthy controls and 3 patients with LN. Cells were stained with FITC-conjugated Live/Dead Fixable Dye (Molecular Probes, Eugene, OR, USA) for 15 min at 4°C in the dark, and subsequently labeled with PE-Cy7-conjugated anti-human CD19 Antibody (Biolegend). After fixation and permeabilization, cells were stained with PerCP-Cy5.5-conjugated anti-human T-bet (Thermofisher, MA, USA) for 30 min at RT. Intracellular T-bet expression was measured in alive cells (detected as FITC^dim^) by flow cytometry, using a BD-FACS Canto instrument, and it was analyzed with Kaluza. The effect of LN-NETs on T-bet expression was evaluated in enriched naïve B lymphocytes, derived from HDs. Cells were cultured in complete medium and stimulated with 2.5 μg/ml CpG2006 and 1.5 μg/ml F(ab')_2_ anti human IgM, IgA, IgG, or only with NET extracts, for up to 48 h, T-bet expression was detected after 48 h stimulation (T48) and compared with the baseline (T0), prior the addition of stimuli. Negative isotype controls were also included in each experiment.

### Software and Statistics

For statistical analysis, GraphPad Prism (Graph Pad software, La Jolla, CA, USA.) software was used. Mann-Whitney U test was used for comparing groups. Statistical significance was defined as *P*-value < 0.05.

## Results

Among 13 SLE patients, enrolled in this study, all except one developed LN during their clinical course. Patients treated with B cell depletion therapy were excluded. At the time of their recruitment 3 patients showed no disease activity (SLEDAI = 0) on low dose glucocorticoids, hydroxychloroquine and mofetil mycophenolate, 6 showed only minimal activity (SLEDAI = 1–5) and the remaining 4 presented a moderate to very high disease activity.

### Uptake of LN-NET by Human B Lymphocytes

**Figure 1:**
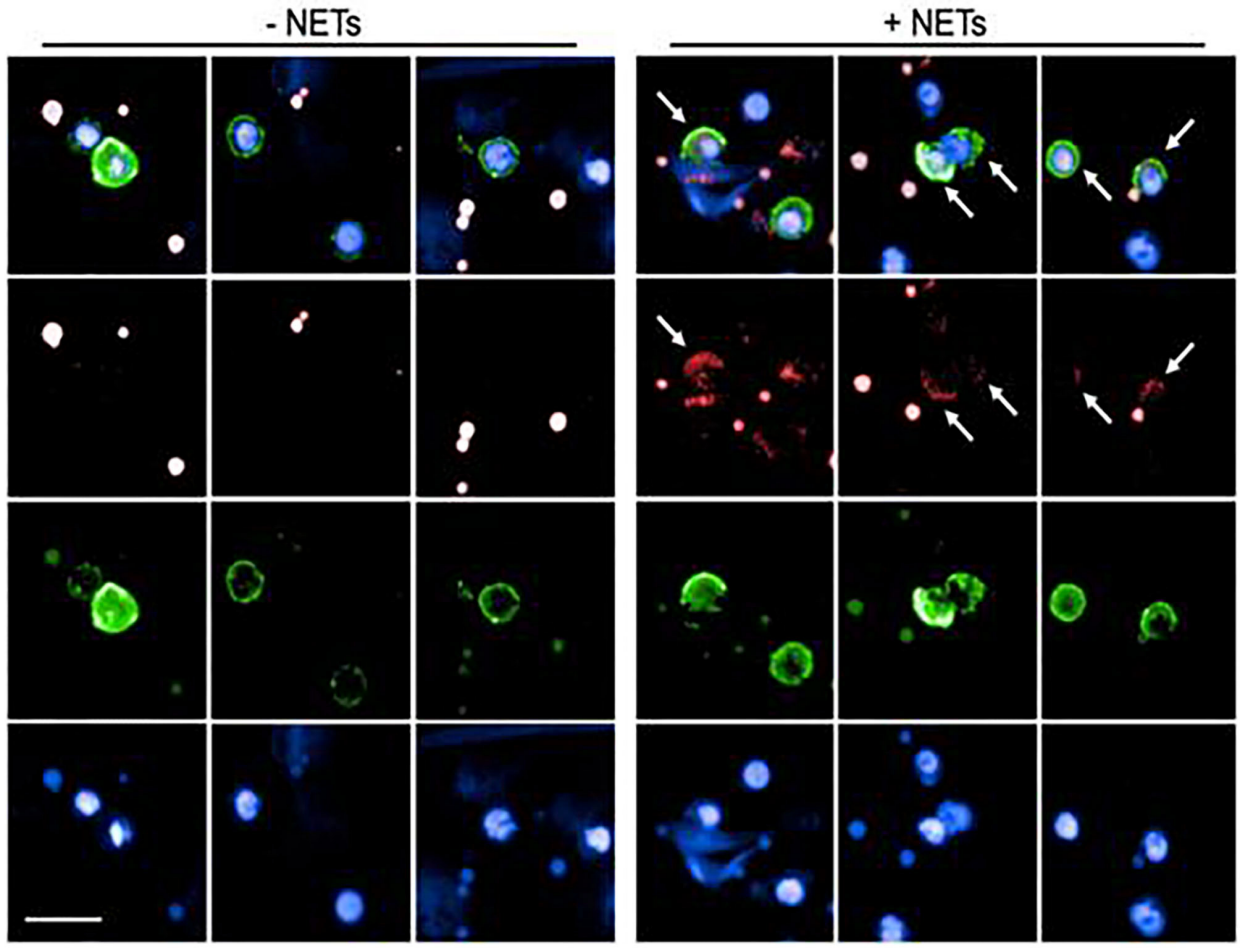
Analysis of NET uptake by B lymphocytes. Representative images of B lymphocytes in the presence (right panel) or absence (left panel) of NET extracts. Surface green staining identifies viable CD20+ B cells. Nuclei were counterstained with Hoechst 33342 (blue staining). NETs were labeled with Alexa 647 conjugated anti-Myeloperoxidase (red staining). Both in the left (untreated cells) and in the right (NET-treated cells) panels, red dots are visible, due to aspecific anti-MPO staining in dead cells. Viable cells, characterized by the integrity of cell membrane (green stain) were analyzed for the presence of small red spots (detectable only in NET-treated cells), indicating specific MPO-staining, and demonstrating the intracellular presence of NET fragments. Arrows indicate the presence of internalized NET fragments.

In order to assess if NETs are internalized by B lymphocytes, that is a prerequisite for activating intracellular signaling pathways, CD20+ B cells, cultured in the presence of anti-Ig and CpG2006 were exposed to NET fragments, obtained by PMA-stimulated neutrophils. As assessed by agarose gel electrophoresis, the molecular size of NET fragments ranged from 100 to 200 kB (Supplementary Figure 2), accordingly to previous observations (20). Myeloperoxidase staining was used to visualize NETs (28). As shown in Figure 1, NETs uptake was evidenced by the presence of intracellular small granules, detected only in viable CD20+ B cells: this pattern was not observed in CD20+ B cells, that were not exposed to NETs.

### LN-NET Does Not Alter “*in vitro*” Proliferation Nor Differentiation of Naïve B Cells

In a preliminary context, we tested the ability of our enriched HD and SLE naïve B cells to proliferate and differentiate into plasmablasts, in response to canonical agents, that are known to trigger class switch recombination (CSR); in parallel, we evaluated the influence of LN-NET exposition on naïve B cells activation. Flow cytometric analysis revealed that, following stimuli, enriched naïve B cells significantly proliferate and differentiate into CD27+ CD38^high^ plasmablasts; the rate of cell proliferation and the percentage of CD27+ CD38^high^ cells were not modified by NET addition (Supplementary Figure 3).

### LN-NET Stimulates IgG2 Production in SLE Naïve B Cells

**Figure 2:**
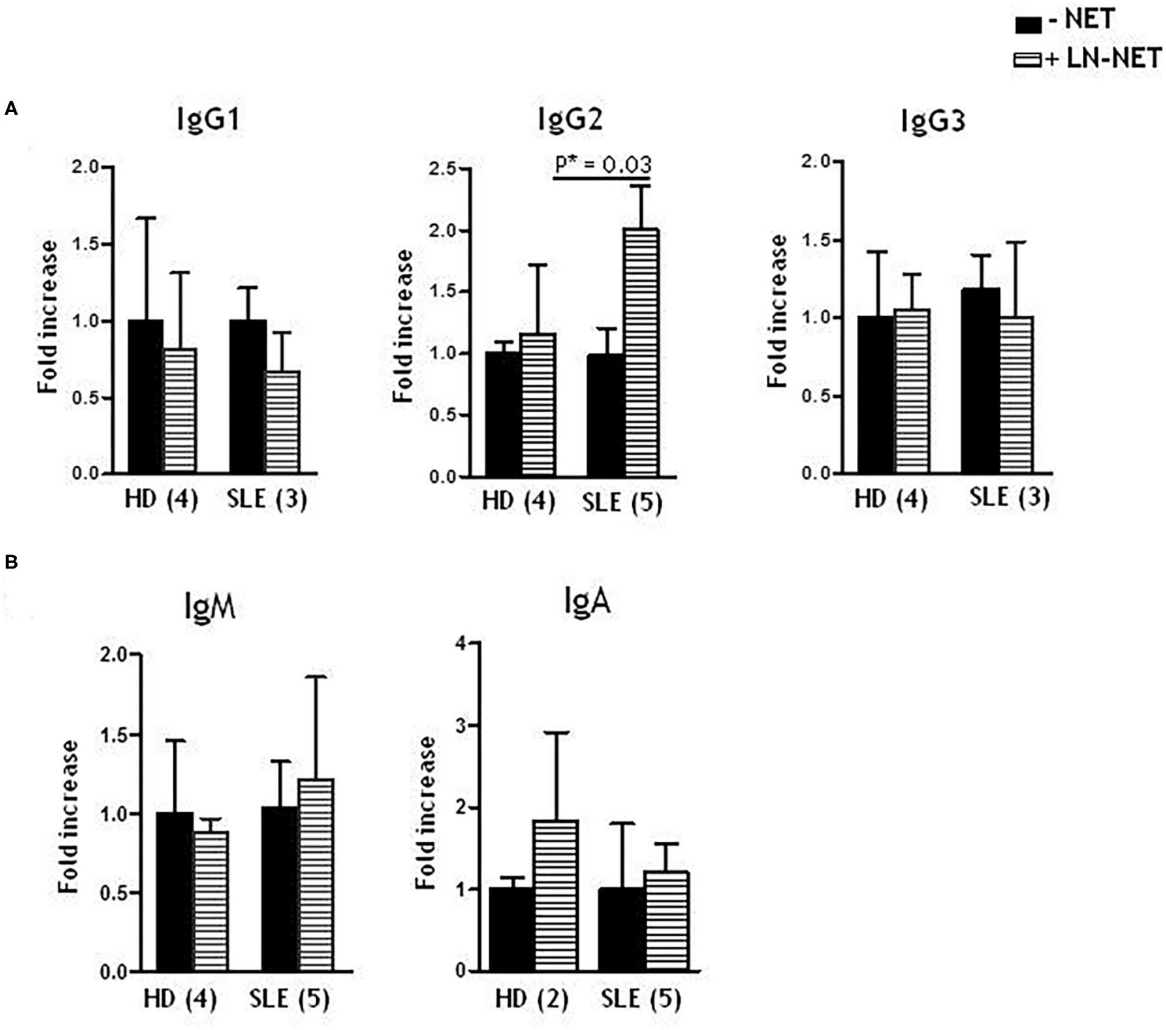
Effect of NET on soluble IgG isotypes, IgM and IgA production. Naïve B cells, derived from healthy donors (HD) or SLE patients, were cultured as described in Materials and Methods. Where indicated (dashed bars), NET extracts were added after 5 days of culture. Immunoglobulins levels were determined by ELISA assay on cell supernatants, collected after 7 days of culture. Values indicate the fold increment of IgG1, IgG2, IgG3 **(A)**, IgM, IgA **(B)**, elicited by NET treatment, vs. untreated cells, and represent the mean of independent experiments indicated in figure.

We analyzed the effect of LN-NET on different immunoglobulin production from enriched, stimulated naïve B cells. We found that soluble IgG2, after LN-NET addition, were significantly increased in SLE naïve B cells supernatants; conversely, this increment was not observed for IgG1 and IgG3 (Figure 2A), as well as IgM and IgA levels (Figure 2B), neither in SLE, nor in normal B cells. We also did not detect a significant increase in IgG2 production by normal naïve B cells, following NET exposition: therefore, the ability of LN-NET to raise soluble IgG2 levels appears to be specific for SLE B cells, By analyzing the responsiveness of individual patients, we did not observe a significant correlation with disease severity (evaluated by SLEDAI score) on IgG2 induction by LN-NET (Supplementary Table 1): for this reason, it is conceivable that LN-NETs exert a pathogenic function in the early phases of autoimmunity, irrespectively of the disease evolution.

### T-Bet Expression Is Stimulated by LN-NET

**Figure 3:**
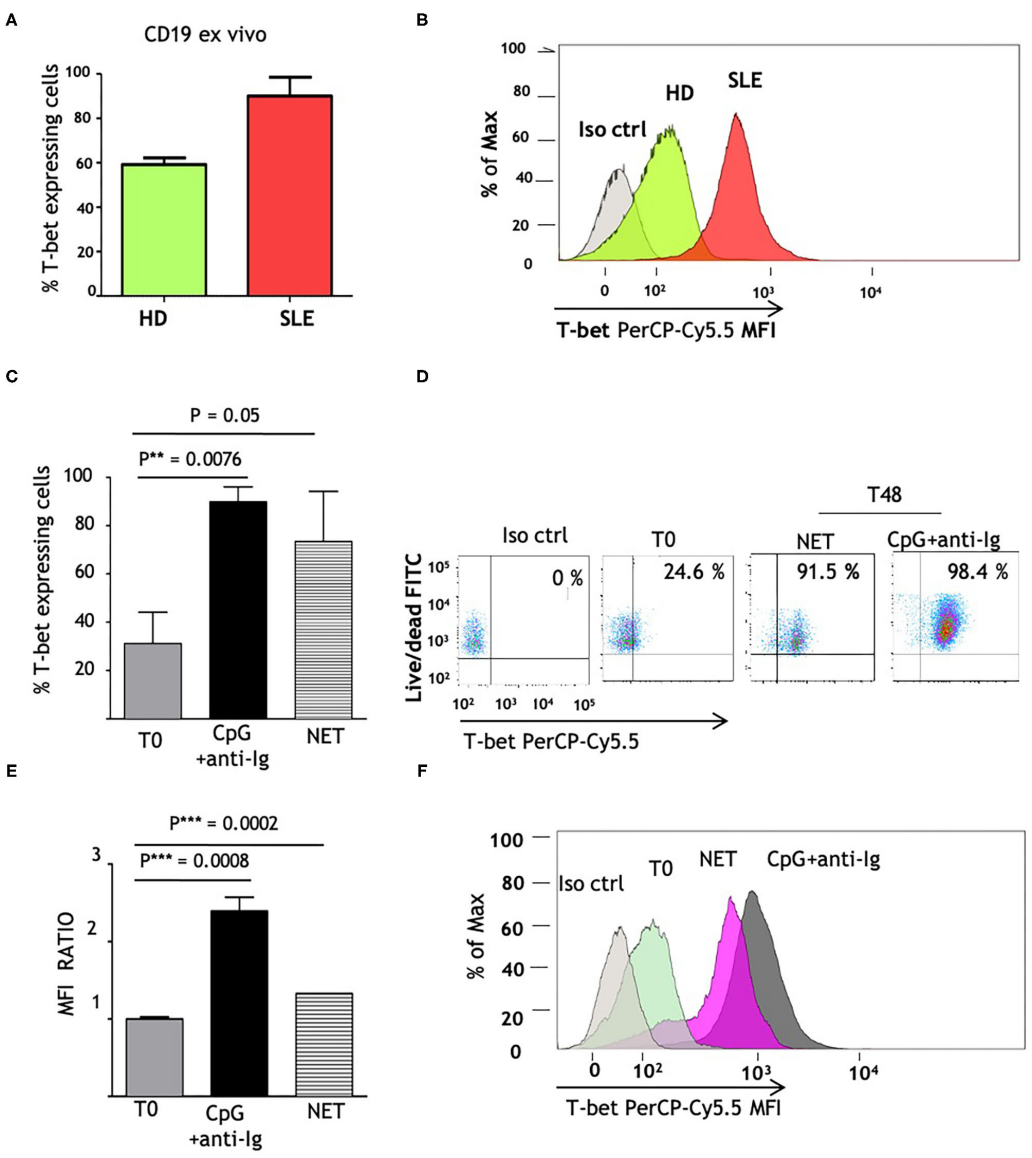
NET induces T-bet expression in human naïve B cells. **(A)** Comparison of *ex vivo* T-bet expression in B lymphocytes, isolated from normal (HD) vs. SLE patients. CD19+ cells were purified from frozen PBMCs samples, as described in section Materials and Methods. Intracellular PerCp Cy5.5 fluorescence (T-bet expression) was assessed by flow cytometry. Values indicate the percentages of PerCp Cy5.5 positive (T-bet expressing) cells, determined in a in gated viable (FITC^dim^) cell population, and represent the mean of three independent experiments. **(B)** Representative overlay histogram depicting PerCp-Cy5.5 Geometric MFI in T-bet expressing cells, detected in one normal control and in one patient with SLE. **(C)** Percentages of T-bet expression in freshly isolated naïve B cells, obtained from healthy donors as described in Materials and Methods, before (T0) and after 48 h stimulation (T48) with CpG+anti-Ig (solid bar), or with LN-NET (dashed bar). NET extracts were added at the concentration of 0,5 μg/ml protein T-bet expressing cells percentages were determined by flow cytometry as described above. The values represent the mean of three independent experiments. **(D)** Representative dot plot of T-bet expression in viable naïve B cells from a healthy donor, in the absence (T0) or presence of different stimuli (as indicated). Upper right quadrants in the graph display T-bet expressing cells. **(E)** Determination of T-bet expression, measured by Geometric Mean Fluorescence Intensity (MFI). Values indicate the fold increment of fluorescence in normal naïve B cells, stimulated for 48 h (T48) with CpG+anti-Ig (solid bar), or with LN-NET (dashed bar), vs. unstimulated (T0) cells, and represent the mean of three independent experiments. **(F)** Representative overlay histogram depicting T-bet expression in B lymphocytes cultured in absence (T0) or presence of indicated stimuli.

Since T-bet is a master regulator of autoreactive B cell differentiation in murine models and is able to stimulate IgG2 class switch (24), we investigated whether its constitutive expression is altered in SLE patients. We analyzed the amount of CD19+ B cells expressing T-bet, that reached a maximum level in SLE patients (>90% of T-bet expressing cells) and was higher than normal controls (Figures 3A,B). We postulated that the elevated T-bet levels, observed in SLE patients, could result from the *in vivo* chronic stimulation of NET and hence we investigated if normal naïve B cells might express T-bet protein to the same extent as SLE B cells, when exposed to LN-NET. We therefore tested the expression of T-bet, before (T0) and after 48 h (T48) of NET stimulation; as a positive control, cells were stimulated with CpG2006 and anti-Ig, powerful inducers of T-bet expression. The percentage of T-bet expressing cells was not elevated in normal controls (Figure 3C; mean = 31.18%); as expected, CpG2006 and anti-Ig induced a robust proliferation coupled with high increase in T-bet expression (mean: 89.9%; *p* = 0.0076). Notably, the addition of NETs from LN patients was sufficient to markedly increase the number of T-bet expressing cells (mean = 73.4%; *p* = 0.08), despite a small effect on cellular proliferation (Figures 3C,D). Consistently, the amount of T-bet protein, measured by Mean Fluorescence Intensity (MFI) detection, was significantly (*p* = 0.0002) induced after stimulation with LN-NET and showed a 30% increase compared to its baseline (T0) expression (Figures 3E,F).

Taken together, these results show that NET from LN patients exerts a direct and specific effect on IgG2 isotype switch in SLE naïve B cells, and also point to T-bet as a putative mediator in this mechanism.

## Discussion

The first finding of this study is the significant increase in IgG2 production, observed in enriched naïve B cells, stimulated with NETs: this effect is selective for IgG2, since other immunoglobulin isotypes are not significantly altered in the same experimental conditions; it is also specific for SLE, as B cells, isolated from normal donors, are unresponsive to NET stimulation. These observations extend the previous findings by Gestermann et al. (20), who showed upregulation of total IgG by memory B cells, after exposure to NETs.

This finding contributes to explain the IgG2 isotype prevalence in autoantibodies of SLE and, in particular, of Lupus Nephritis patients, and propose NET as a culprit in these conditions.

The exclusive responsiveness of SLE patients also suggests that a pre-existing condition predisposing to autoimmunity is necessary to amplify the effects of NET on IgG2 production: in this regard, recent investigations highlight an epigenetic priming in naïve B cells of SLE patients, with respect to their counterparts in healthy donors (29).

Our results, on the other hand, indicate that NET does not come into play in the first phases of class switch recombination (CSR), characterized by cellular proliferation and generation of plasmablasts, and it is rather conceivable that NETs activate subsequent steps that are crucial for the preferential skewing of defined IgG subclasses: in this respect NETs would interact and cooperate with specific cytokines and transcriptional factors, able to tailor a context-specific immune response (30).

We therefore focused on T-bet transcription factor, as a potential key mediator in the IgG2 induction, elicited by NETs. Experimental murine models previously clarified the role of T-bet in IgG2a production (31, 32); moreover, T-bet is the hallmark of the potentially autoreactive B lymphocyte compartment, referred as “atypical memory” (22) or “naïve activated” (33) cells, highly expanded in active SLE patients with LN. In this cell subset, T-bet expression is induced by inflammatory Th1 cytokines and, interestingly, this occurs prior to their differentiation into Antibody-Secreting Cells (22). We postulated that NETs accumulation in SLE (34) could create an inflammatory milieu (35), that would drive the formation of an autoreactive, T-bet^high^ B cell pool, prone to generate IgG2 autoantibodies: this idea seems to find a confirmation in the overexpression of T-bet, that we observed in B lymphocytes of SLE patients. A further evidence about a link between NET and T-bet derives from our results from normal enriched naïve B cells, that constitutively express T-bet from low to moderate levels: indeed, we demonstrated that, in the absence of canonical inducing factors, separately evaluated as positive controls, lupus NET fosters T-bet expression.

This is the first observation concerning the direct ability of NETs to upregulate T-bet in human naïve B cells. Given the multiple functions orchestrated by T-bet in the immune response (36), this finding strengthens the concept of NET as a crucial player in the development of SLE auto-reactivity. T-bet is indeed expressed by a wide number of immune cells and is critical for the differentiation of professional, tissue-resident memory T cells, as well as T follicular helper (Tfh) and Th17 cells. In B lymphocytes, the cooperation of T-bet with an additional transcription factor, Bcl-6, enables the formation and survival of autoreactive germinal centers (GCs), that are specialized sites for the selection of high affinity-responsive B cells (26, 31). Therefore, T-bet could play an important role in the relocation of cells from secondary lymphoid organs to ectopic GCs, able to generate autoreactive plasmablasts.

Besides these direct effects, T-bet mediates the expression of CXCR3, a specific homing chemokine receptor responsible for the migration to inflamed tissues of T and B lymphocytes, and thus promotes the co-localization of multiple cell types, with different effector functions, in target organs (37). Particularly, in the kidney, during LN development, the migration of CD4+, CD8+, and IL17-producing T cells drives the formation of an autocrine, inflammatory compartment. Notably, polymorphonuclear cells may also be recruited in the inflamed kidney and pushed to undergo NETosis: this creates a vicious cycle, responsible for the uncontrolled amplification of the autoimmune response (38).

Further investigations are required to identify the mechanisms triggered by NET and involved in T-bet induction. T-bet is known to be upregulated in mice by hypomethylated CpG through TLR9 ligation (39): nonetheless, owing to the low expression of this receptor in naïve B cells (40), we can hypothesize that BCR could exert a predominant function in NET signaling. BCR engagement acts in synergism with TLR9 (41, 42) and, intriguingly, its ligation increases TLR9 expression in naïve B cells (43). More studies are necessary to clarify this point. Additional factors, including type I IFN, IRF5 (44, 45), or MyD88-related pathways (46), that could cooperate with NET in T-bet induction, should also be considered. In particular, the identification of signals that might be specifically activated by the peculiar protein components of LN-NETs is still lacking and represents an exciting challenge.

Moreover, the mechanisms underlying the hyperresponsiveness of SLE naïve B cells to NET need to be examined in depth. In this regard, it is known that, besides T-bet, other transcription factors are implicated in ASC differentiation (47), and their relative concentration is thought to influence significantly B cell fate in different physiological or pathological conditions. Regulatory factors antagonizing T-bet, such as the zinc finger transcription factor Ikaros (48) and c-Myb (49), could have a substantial function in healthy subjects and might justify the negative effect of NETs in normal B cells.

In conclusion, further research is needed to better define the role of T-bet in the complex network that tightly regulates CSR and IgG isotype switch (42). Our work reinforces the key role of NET in autoimmunity and propose NETosis as a target for new therapeutic protocols (50), in the treatment of SLE and Lupus Nephritis.

## Data Availability Statement

The original contributions presented in the study are included in the article/Supplementary Material, further inquiries can be directed to the corresponding author/s.

## Ethics Statement

The studies involving human participants were reviewed and approved by Internal Ethic Committee, IRCCS Giannina Gaslini Institute. The patients/participants provided their written informed consent to participate in this study.

## Author Contributions

RB: design of the study, most of experiments and analysis, data interpretation, manuscript writing, and final approval. FS: experiments and data analysis, data interpretation, manuscript writing, and final approval. SS: coordination of patients' samples collection, clinical data collection, and elaboration of manuscript. NP: confocal analysis. FA and DR: cell sorting experiments. AC, SN, and FP: coordination of patients' samples collection, provision of patients' material, and clinical data collection. MG: provision of patients' material, financial support, elaboration of the manuscript, and final approval. SV: design of the study, data interpretation, financial support, critical revision of the manuscript, and final approval of manuscript. GMG: design of the study, data interpretation, financial support, critical revision of the manuscript, and final approval of manuscript.

## Conflict of Interest

The authors declare that the research was conducted in the absence of any commercial or financial relationships that could be construed as a potential conflict of interest.
