# Supplementary material for: Neutrophil Extracellular Traps in Systemic Lupus Erythematosus Stimulate IgG2 Production From B Lymphocytes
Source: Front Med (Lausanne). 2021 Apr 12;8:635436. doi: 10.3389/fmed.2021.635436 (PMC8072216; doi:10.3389/fmed.2021.635436)

Supplementary Figure 1. Gating strategy for naive B cells sorting. Freshly isolated CD19+ cells were stained with anti-human CD19 PE-Cy7, CD24 FITC and CD38 APC-conjugated monoclonal antibodies. Cell sorting was performed with a FACS Aria instrument; CD19+ cells were > 90% of total; naïve B cells (CD24-, CD38 ^{low}) were sorted on gated CD19+ cells, and accounted for approximately 60% of CD19+ cell population

Fig.1 Suppl.

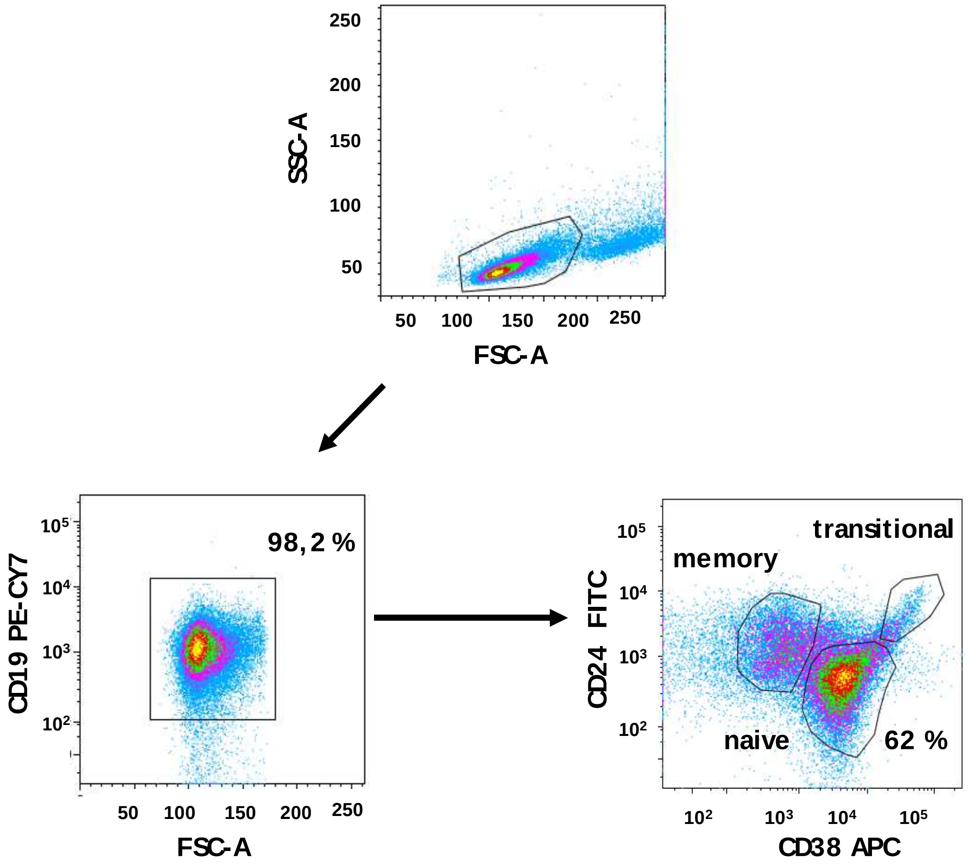

Supplement: Supplementary file 1 [file Image_1.pdf]
