# Supplementary material for: Neutrophil Extracellular Traps in Systemic Lupus Erythematosus Stimulate IgG2 Production From B Lymphocytes
Source: Front Med (Lausanne). 2021 Apr 12;8:635436. doi: 10.3389/fmed.2021.635436 (PMC8072216; doi:10.3389/fmed.2021.635436)

Supplementary Figure 2. Determination of molecular weight (MW) of NET fragments. NETosis was induced in Lupus PMNs by PMA stimulation, as described in Methods. NET extracts were then mildly digested with Micrococcal DNase and the resulting fragments were analyzed by 2% agarose gel electrophoresis.

Fig.2 Suppl.

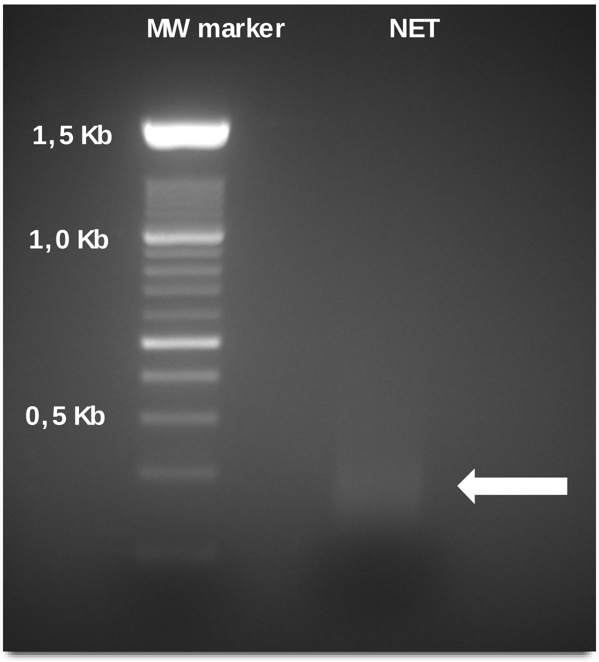

Supplement: Supplementary file 2 [file Image_2.pdf]
