# Supplementary material for: Neutrophil Extracellular Traps in Systemic Lupus Erythematosus Stimulate IgG2 Production From B Lymphocytes
Source: Front Med (Lausanne). 2021 Apr 12;8:635436. doi: 10.3389/fmed.2021.635436 (PMC8072216; doi:10.3389/fmed.2021.635436)

Supplementary Figure 3. Dot plot (one out of four independent experiments), representing the effect of SLE-NET on naive B cell phenotype, proliferation and differentiation in vitro.
Flow cytometric analysis in viable (PI negative) CD19+ cells of CD27, CD38 markers and proliferation rate assessment by CFSE dilution assay. Naive B cells were stimulated for 7 days with CpG and anti-Ig, without NET (A) or with SLE NET(B)

A

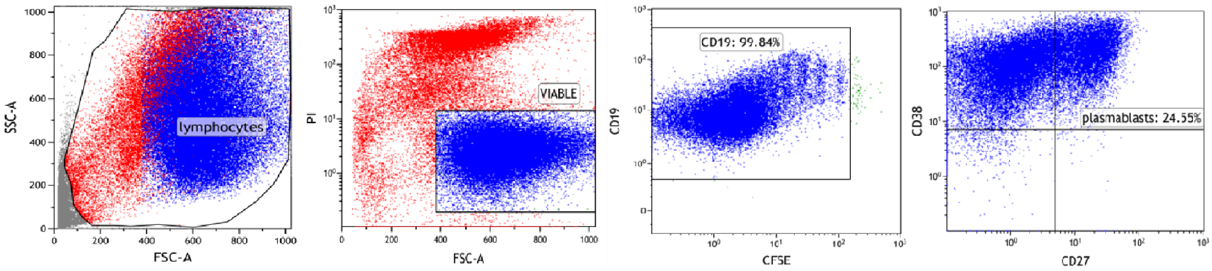

B

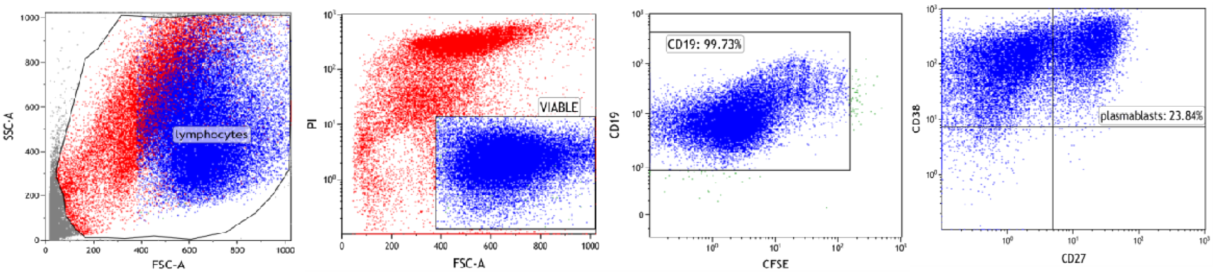

Supplement: Supplementary file 3 [file Image_3.pdf]
