# Supplementary material for: Neutrophil Extracellular Traps in Systemic Lupus Erythematosus Stimulate IgG2 Production From B Lymphocytes
Source: Front Med (Lausanne). 2021 Apr 12;8:635436. doi: 10.3389/fmed.2021.635436 (PMC8072216; doi:10.3389/fmed.2021.635436)
Supplement: Supplementary file 4 [file Table_1.docx]

**Supplementary Table 1. Clinical features of SLE patients**

| Pt | Sex | Age at evaluation | Age at disease onset | Disease onset symptoms and laboratory tests | Nephritis during clinical course/ Class | Symptoms and laboratory features at evaluation | Autoantibodies at evaluation | Treatment at evaluation | SLEDAI-2K at the evaluation |
| --- | --- | --- | --- | --- | --- | --- | --- | --- | --- |
| 1 | F | 16y 5 mo | 16y 1mo | Arthritis, leukopenia, malar rash, oral ulcers, reduced complement,raised ESR, positive anti ds DNA antibodies, ANA > 1.320, persistent proteinuria, urinary cellular casts, hypergammaglobulinemia | Yes/ IV-V | Oral ulcers, Nephritis (class IV-V), proteinuria, pyuria, haematuria | Anti ds DNA positive (884) | Prednisone 1 mg/kg, Cyclophosfamide iv 500 mg (one infusion) | 20 |
| 2 | F | 15y 10 mo | 11y 5 mo | Arthritis, malar rash, reduced complement,raised ESR, positive anti ds DNA antibodies (486), ANA > 1.320 | Yes/V | Asymptomatic | Anti ds DNA positive (95), ANA 1:80 | HHQ, MMF, prednisone 3.75 mg/day | 2 |
| 3 | F | 18y 9 mo | 14y | Fever, arthritis, pericarditis, pleuritis,malar rash, raised ESR, positive anti dsDNA antibodies, ANA 1:320 | Yes/IIIA | Asymptomatic | Anti ds DNA negative, ANA 1:160 | HHQ, MMF, prednisone 2.5 mg/day | 0 |
| 4 | F | 17y 9 mo | 9 y 6 mo | Fever, arthritis, raised ESR, positive anti ds DNA antibodies,pericarditis, ANA 1:640,reduced complement, positive aCL and LAC | Yes/IV | Asymptomatic | Anti ds DNA negative, ANA negative | HHQ, MMF, prednisone 2.5 mg/day | 0 |
| 5 | F | 21y | 13y 10 mo | Fever, arthritis, malar rash, raised ESR, positive anti ds DNA antibodies, ANA 1:320, reduced complement, positive aCL, anti beta2 glycoprotein I antibodies positive | Yes/III | Reduced complement, lymphopenia | Anti ds DNA positive (111), ANA >1:320, positive aCL | HHQ, MMF, prednisone 5 mg/day, low dose ASA | 4 |
| 6 | F | 19y 10 mo | 13y 9mo | Arthritis, malar rash, raised ESR, positive anti ds DNA antibodies, positive ANA,reduced complement, positive aCL, anti beta2 glycoprotein I antibodies positive and LAC, inferior limbs bilateral arterial thrombosis (antiphospholipid syndrome) | No | Reduced complement | Anti ds DNA negative, ANA 1:80 | HHQ, MMF, prednisone 5 mg/day, low dose ASA | 2 |
| 7 | M | 14y 9mo | 14y 2mo | Fever, malar rash, raised ESR, ANA 1:160 positive anti ds DNA antibodies, ,reduced complement, positive aCL, positive LAC, anemia, autoimmune thrombocytopenia,hematuria, raised creatinin. | Yes/II | Raised ESR, reduced complement, anemia, autoimmune thrombocytopenia, nephritis. | ANA 1:160,anti ds DNA negative, positive aCL, positive LAC, | Prednisone 0.5 mg/kg | 11 |
| 8 | F | 14 y 10 mo | 12 y | Arthritis, malar rash, oral ulcers, reduced complement, positive anti ds DNA antibodies, positive ANA, nephritis | YES/IV | Reduced complement | ANA 1:320,anti ds DNApositive 1:80, negative aCL | MMF, prednisone 5 mg/day | 4 |
| 9 | M | 24 y | 6 y | Arthritis, anemia, nephritis | YES/V | Raised ESR, persistent proteinuria, haematuria, hypertension, raised creatinin | Negative aCL and LAC | MMF, prednisone 10 mg/day | 8 |
| 10 | F | 17y 9mo | 10y 6mo | Arthritis, fever, skin vasculitis, reduced complement, positive anti ds DNA antibodies, positive ANA, nephritis | YES/IV | Asymptomatic | ANA negative,anti dsDNA positive | HHQ,MMF, prednisone 1.25 mg/day, | 2 |
| 11 | F | 41y 6mo | 28y | Pleuro-pericardic effusion, pericarditis, positive Coombs test, persistent proteinuria, positive ANA, reduced complement | YES/IV | Optic neuritis | Anti ds DNA negative, ANA 1:320, aCL IgM and IgG positive, LAC positive | HHQ | 8 |
| 12 | F | 49y | 45y | Fever, skin rash, lymphonodes enlargement, pericardial effusion, positive ANA, proteinuria, reduced complement, pancytopenia | YES* | Arthralgia, reduced complement | anti dsDNA negative | HHQ, MTX, Belimumab, Prednisone 5 mg/day | 4 |
| 13 | F | 57y | 25y | Arthralgia, fever, positive anti ds DNA antibodies, positive ANA, nephritis | YES* | Asymptomatic, mild proteinuria (<0.5g/24h) | ANA negative | Off therapy | 0 |

* no histological details available

aCL-anti cardiolipin antibodies; ANA-anti nuclear antibodies; anti dsDNA-anti double strand DNA antibodies; ESR-erythrosedimentation rate; HHQ- hydroxychloroquine; LAC- Lupus anti coagulant; MMF- mycophenolate mofetil , MTX-Methotrexate
